# First survey on association of *TMEM154* and *CCR5* variants with serological maedi-visna status of sheep in German flocks

**DOI:** 10.1186/s13567-018-0533-y

**Published:** 2018-04-19

**Authors:** Vahid Molaee, Marwa Eltanany, Gesine Lühken

**Affiliations:** Department of Animal Breeding and Genetics, Justus Liebig University of Giessen, Ludwigstrasse 21, 35390 Giessen, Germany

## Abstract

Maedi-visna, a disease caused by small ruminant lentiviruses (SRLVs), is present in sheep from many countries, also including Germany. An amino acid substitution (E/K) at position 35 of the transmembrane protein 154 (*TMEM154*) as well as a deletion in the chemokine (C-C motif) receptor type 5 gene (*CCR5*) were reported to be associated with the serological MV status and/or the SRLV provirus concentration in North American sheep populations. The aim of this study was to test if those two gene variants might be useful markers for MV susceptibility in Germany. For this purpose, more than 500 sheep from 17 serologically MV positive German sheep flocks with different breed backgrounds were genotyped applying PCR-based methods. Both, crosstab and non-parametric analyses showed significant associations of the amino acid substitution at position 35 of *TMEM154* with the serological MV status (cut-off-based classification) and the median MV ELISA S/P value in all samples and in two of the four analyzed breed subsets. The deletion in the *CCR5* promoter did not show a consistent association with serological MV status or median ELISA S/P value. It can be concluded that the amino acid substitution at position 35 of *TMEM154* is a promising marker for breeding towards a lower number of serologically MV positive sheep in German flocks, at least in flocks of the Texel breed, while this remains questionable for the deletion in the *CCR5* promoter. The findings of this study still need to be verified in additional sheep breeds.

**Electronic supplementary material:**

The online version of this article (10.1186/s13567-018-0533-y) contains supplementary material, which is available to authorized users.

## Introduction

Small ruminant lentiviruses (SRLVs) belong to the family *Retroviridae* and cause diseases called maedi-visna (MV) in sheep and caprine arthritis encephalitis (CAE) in goats. Maedi-visna is widespread in sheep from many countries around the world. No European country except for Iceland can be considered to be free of SRLV infection [1]. The disease is also substantially spread among sheep flocks in Germany [2]. There is no cure for the chronic disease caused by SRLVs, which includes symptoms such as pneumonia, wasting, mastitis, arthritis and progressive paralysis [3]. A vaccine preventing SRLV infection has not been developed yet [4]. Production losses stem from lamb mortality, lower lamb weights and milk production from older infected ewes [5, 6], early culling [1] and export restrictions [7]. The significant economic losses have led to the development of control programs in Europe and elsewhere which commonly include separation of lambs from dams at birth to prevent virus transmission and test/cull methods. Although they can be successful [1], these methods are neither cost-effective [8] nor sustainable, as SRLV-free flocks are still susceptible to infection if exposed to other infected sheep or goats [9].

Due to these limitations of conventional strategies for the control of SRLV infections, a genetically based approach would be favorable. However, significant evidence for host genetic variation of resistance/susceptibility is a basic requirement for such a strategy [10]. Indeed, there is a confirmed genetic predisposition to resist SRLV infection at different levels among and within breeds [11–14].

The apparent differences in MV susceptibility between sheep breeds led to studies on possibly associated host genetic variation and finally to the detection of variants in several genes (e.g. *TMEM154*, *CCR5*, *MHC*, *ZNF389*, *TLRs* and *APOBEC3*) showing association with parameters of MV susceptibility [15–17]. Specifically, variants in the genes coding for transmembrane protein type 154 (*TMEM154*) and chemokine (C-C motif) receptor type 5 (*CCR5*) have been reported to be associated with the serological status and/or the provirus concentration of sheep in MV affected US sheep flocks [18, 19].

After the initial discovery of a possible involvement of *TMEM154* in the genetic control of MV susceptibility by genome-wide association studies, several polymorphic positions were identified in the coding region of this gene. Two *TMEM154* haplotypes, both carrying a nucleotide coding for glutamate (E) at amino acid position 35 of the *TMEM154* protein were associated with susceptibility to SRLV infection (as determined by the serological MV status of sheep), whereas a third haplotype, carrying a nucleotide coding for lysine (K) at position 35 was not [19]. This association was confirmed in additional cohorts in the same and in other studies [20, 21], but to our knowledge until now has not been tested in sheep populations outside of North America.

Approaching potential genetic factors for MV susceptibility from a different point of view, existing data in diverse species indicated that *CCR5* could be a candidate gene for resistance to various pathogens [16, 22–27]. Even more important in the context of this study is that humans carrying *CCR5* variants seem not to acquire human immunodeficiency virus (HIV) [28–30], the best-explored lentivirus. Based on this knowledge, White et al. [18] analyzed the *CCR5* variation in sheep and tested a 4-base promoter deletion for association with proviral levels of SRLV in sheep. Individuals carrying two copies of the mutant variant (deletion) had significantly reduced proviral levels [18].

Thus, the amino acid substitution at position 35 of *TMEM154* as well as the *CCR5* promoter deletion are promising candidates as selection tools to decrease MV susceptibility in sheep. However, once a genetic association with pathogen resistance has been detected in a population and/or country, the practical relevance of the identified genetic marker(s) cannot be generalized. In other populations and/or countries, variations in pathogen strains, host breeds, and environmental conditions may impair the effect of the identified variant(s). In this respect, the repeated testing in different animal sets is necessary and can confirm or disprove a proposed candidate gene [17]. Therefore, the aim of this study was to test if the amino acid substitution at position 35 of *TMEM154* as well as the *CCR5* promoter deletion could be useful markers for selection against MV susceptibility in the German sheep population. We report the results of the analysis of their association with the serological MV status and median MV ELISA S/P value of more than 500 sheep from 17 MV positive German sheep flocks with different breed backgrounds.

## Materials and methods

### Collection of animal samples

A total of 656 sheep from 23 German sheep flocks were sampled between 2014 and 2016 to evaluate the serological MV status. From the oldest ewes (minimum age was 4 years) of each flock whole blood samples were drawn from the jugular vein into 9 mL EDTA monovettes. In order to get a higher number of samples, in one flock (which was of special interest because it contained two pure breeds), samples were collected from all ewes with a minimum age of 3 years (few samples from even younger ewes) and also from rams older than 4 years. The sampled sheep flocks were located in the German states Schleswig-Holstein, Nordrhein-Westfalen, Mecklenburg-Vorpommern, Hessen and Baden-Württemberg. Only samples from flocks with serologically MV positive sheep were genotyped and included into association analyses, and divided into four sample sets (breed subsets) according to their breed background.

### Serological testing for MV status

The plasma was separated by centrifugation at 3000* g* for 10 min and stored at −20 °C. Plasma samples were shipped to the laboratory of the Animal Health Service of Thüringen (Jena, Germany) for serological testing with an enzyme-linked immunosorbent assay (ELISA) (IDEXX CAEV-MVV Total Ab ELISA, IDEXX GmbH, Ludwigsburg, Germany), according to the manufacturer’s instructions. This ELISA is one of three ELISA tests officially approved for SRLV diagnosis in sheep and goats by the German licensing authority [Friedrich-Loeffler-Institute (FLI), Federal Research Institute for Animal Health, Island of Riems, Germany].

According to the guidelines of the used ELISA kit, the cut-off value is defined based on the corrected optical density (OD) at a wavelength of 450 nm ratio of sample to positive control (S/P):

$${{\text{S}} \mathord{\left/ {\vphantom {{\text{S}} {\text{P}}}} \right. \kern-0pt} {\text{P}}} = 100 \times \frac{{{\text{mean}}\;{\text{OD}}\; 450\;{\text{value}}\;{\text{of}}\;{\text{the}}\;{\text{sample}} - {\text{mean}}\;{\text{OD}}\;450\;{\text{value}}\;{\text{of}}\;{\text{the}}\;{\text{negative}}\;{\text{controls}}}}{{{\text{mean}}\;{\text{OD}}\;450\;{\text{value}}\;{\text{of}}\;{\text{the}}\;{\text{positive}}\;{\text{controls}} - {\text{mean}}\;{\text{OD}}\;450\;{\text{value}}\;{\text{of}}\;{\text{the}}\;{\text{negative}}\;{\text{controls}}}}$$

Samples were considered as serologically MV negative with an S/P value ≤ 110% and recorded as positive with an S/P value ≥ 120%. Suspicious results were in the range between 110 and 120% and excluded from further analyses.

### Genotyping of *TMEM154* and *CCR5* variants

After plasma separation, residual blood was stored at −20 °C until extraction of genomic DNA using a modified salting out method [31].

The KASP technology (LGC, Hoddesdon, UK) was used for genotyping a nucleotide substitution in the coding region of *TMEM154* (*Ovis aries* chromosome 17, Oar_v4.0, NC_019474.2:g.4860407G>A), resulting in the substitution of the ancestral glutamic acid (E) with lysine (K) at position 35 of the mature protein. For this purpose, a common forward primer and two allele-specific primers were designed and synthesized by LGC (primer details are given in Additional file 1). Polymerase chain reactions (PCR) including the respective primers, the KASP master mix and 50 ng DNA were set up as recommended by LGC. PCR amplification was done in a Bio-Rad CFX96 thermal cycler (Bio-Rad Laboratories, München, Germany) under the following conditions: initial denaturation at 94 °C for 15 min, 10 touchdown cycles at 94 °C for 20 s, 61–55 °C (decreasing 0.6 °C per cycle) for 1 min, and 26 cycles at 94 °C for 20 s and 50 °C for 1 min, followed by a final cycle at 37 °C for 1 min. Fluorescence measurement and end point allelic discrimination were done in the same instrument and with the included software (CFX Manager 3.1).

Fragment-length analysis was used for determination of the presence or absence of a deletion in the promoter region of *CCR5* (*Ovis aries* chromosome 19, Oar_v4.0, NC_019476.2:g.52961717_52961714delAATG, minus strand). The reverse primer of the selected primer pair was fluorescence-labeled (primer details are given in Additional file 1). PCR reactions were performed in a final volume of 15 μL containing 20–70 ng of template DNA, 10 pmol of each primer, 2 mM dNTPs, 1× Go Taq Flexi PCR buffer and 0.5 units Go Taq Polymerase (Promega, Mannheim, Germany). The following PCR conditions were used: initial denaturation at 95 °C for 1.5 min, followed by 30 cycles at 95 °C for 15 s, 52 °C for 15 s and 72 °C for 15 s, and a final extension at 72 °C for 5 min. Fragment-length analysis of denaturated PCR products was done with an ABI 3130 automated sequencer and the software GeneMapper version 4.0 as recommended by the manufacturer (Applied Biosystems, Darmstadt, Germany).

Both genotyping methods were tested for their accuracy by a comparison of assay-derived genotypes with the results of direct Sanger sequencing. For this purpose, PCR products including the genotyped variants were amplified and sequenced (primer details are given in Additional file 1).

### Statistical analyses

The SPSS program (version 23.0) for Windows (IBM SPSS Statistics, Armonk, NY: IBM Corp) was used for statistical analyses.

The independent segregation of alleles was assessed for Hardy–Weinberg equilibrium (HWE) by using Fisher’s exact test. Differences in the distribution of allele and genotype frequencies between groups of serologically MV negative and positive samples were tested by Fisher’s exact test (when expected values were < 5) or χ^2^ test. Consistent with a dominant effect of the risk allele and a recessive effect of the protective allele, frequencies of genotypes with one or two copies of the putative risk allele (*TMEM154* E, or the wild type variant of the *CCR5* promoter) were combined.

The Shapiro–Wilk’s test [32] was used to check whether ELISA S/P values were normally distributed. ELISA S/P values were described using median and interquartile range (IQR). Independent sample nonparametric analysis using median test was performed to compare ELISA S/P values of groups. For multiple comparisons, the threshold for significance was corrected using Bonferroni’s correction.

The relative risk (RR) to be serologically MV positive (in a MV affected flock) was estimated for animals carrying one and/or two copies of the putative susceptible allele (risk factor) with the method of Altman [33] using the following equation:

$${\text{RR}} = \frac{{{\text{a}}/\left( {{\text{a }} + {\text{b}}} \right)}}{{{\text{c}}/\left( {{\text{c}} + {\text{d}}} \right)}}$$

where *a* is the number of serologically MV positive individuals carrying the risk factor, *b* is the number of serologically MV negative individuals carrying the risk factor, *c* is the number of serologically MV positive individuals carrying no risk factor, and *d* is the number of serologically MV negative individuals carrying no risk factor.

## Results

### Serological MV status of sampled flocks and breed composition of MV positive flocks

A total of 656 samples from 23 flocks, originating from five German states, were sampled and serologically tested for MV status. Samples from 11 sheep with MV ELISA S/P values between 110 and 120% were excluded from all analyses. Details on state of origin, breed composition, sample numbers and percentage of serologically MV positive samples for each flock are given in Additional file 2. In six of these flocks, all tested samples (*n* = 115) were serologically negative. The ELISA S/P values of samples from these six flocks, which were not included in further analyses, ranged from 0.43 to 72.73% with a median of 9.69% and interquartile range from 6.23 to 16.32%.

In contrast, in the other 17 sampled sheep flocks, a minimum of 10% up to 100% of the collected samples showed ELISA S/P values higher than 120%. These flocks were considered to be MV affected and assigned to four breed subsets according to the breed composition (details in Additional file 2). Subset 1 (TEX-x) included samples from 15 different flocks which all consisted of purebred and/or crossbred German Texel sheep (flocks 1–12 and 14–16a). Subsets 2–4 included samples from single flocks each, with purebred German Blackheaded Mutton (GBM) in subset 2, purebred and crossbred Merinoland sheep (MLS-x) in subset 3, and East Friesian milk and Lacaune sheep and the respective cross-breeds (EFM–LAC) in subset 4 (flocks 16b, 22, 23, respectively). The Texel and German Blackheaded Mutton sheep from flocks 16a (part of subset 1) and 16b (subset 2) were kept together.

In all 17 MV affected flocks, ELISA S/P values ranged from −6.34 to 109.65% in the group of serologically negative samples (*n* = 207) and from 121.89 to 328.63% in the group of serologically positive samples (*n* = 323). More descriptive MV-ELISA S/P data, including each breed subset, are given in Additional file 3. The Shapiro–Wilks test for normality showed a significant deviation of the ELISA S/P values from normal distribution in serologically negative and positive sheep in all samples and within breed subsets, except for the small group of serologically positive GBM (subset 2). In positive samples it was right-skewed (medians higher than means) and in negative samples it was left-skewed (medians lower than means).

### Frequencies of putative risk/protective alleles and genotypes of *TMEM154* and *CCR5* in serologically MV positive and negative sheep

In all genotyped samples from 17 MV affected flocks, the putative protective allele (K) and the risk allele (E) at amino acid position 35 of *TMEM154* were observed at frequencies of 48 and 52%, respectively. Deviation from the Hardy–Weinberg equilibrium was only observed for breed subset 3 (MLS-x). In the breed subsets GMB, MLS-x and EFM–LAC the K allele was more frequent (63, 80 and 68%, respectively), whereas in the subset TEX-x it was less frequent (33%) than the E allele.

Comparing groups of serologically MV negative and positive sheep for the *TMEM154* variation, in all samples and in all four breed subsets, frequencies of the protective allele (K) and genotype (KK) were higher in serologically MV negative sheep than in serologically MV positive sheep (Table 1). Vice versa, frequencies of the risk allele (E) and the risk genotypes (EK and EE) were higher in MV positive sheep. These differences between groups of MV negative and positive sheep, either for comparison of the putative protective allele against the risk allele, or the protective genotype against the risk genotypes, were statistically significant in all samples. Concerning breed subsets, this association was significant in TEX-x and EFM–LAC, narrowly missed the significance threshold in MLS-x, and was not significant at all in the GMB subset (Table 1).

**Table 1:** ***TMEM154*** **E/K allele and genotype frequencies in serologically MV positive and negative sheep**

Breed subset	MV status	*TMEM154* allele frequency (n chromosomes)		*P* value	*TMEM154* genotype frequency		*P* value
(n sheep)	(n sheep)				(n sheep)	(n sheep)	
		K	E		KK	EK, EE	
All (527)	Negative (206)	0.65 (268)	0.35 (144)	< 0.001	0.48 (100)	0.52 (106)	< 0.001
	Positive (321)	0.36 (233)	0.64 (409)		0.15 (47)	0.85 (274)	
TEX-x (341)	Negative (95)	0.48 (92)	0.52 (98)	< 0.001	0.30 (29)	0.70 (66)	< 0.001
	Positive (246)	0.26 (130)	0.74 (362)		0.04 (9)	0.96 (237)	
GBM (39)	Negative (35)	0.66 (46)	0.34 (24)	0.140	0.40 (14)	0.60 (21)	0.277
	Positive (4)	0.37 (3)	0.63 (5)		0.00 (0)	1.00 (4)	
MLS-x (125)	Negative (62)	0.85 (105)	0.15 (19)	0.067	0.74 (46)	0.26 (16)	0.067
	Positive (63)	0.75 (95)	0.25 (31)		0.59 (37)	0.41 (26)	
EFM–LAC (22)	Negative (14)	0.89 (25)	0.11 (3)	0.001	0.79 (11)	0.21 (3)	0.006
	Positive (8)	0.31 (5)	0.69 (11)		0.13 (1)	0.87 (7)	

TEX-x: purebred and crossbred German Texel sheep, GBM: purebred German Blackheaded Mutton sheep, MLS-x: purebred and crossbred Merinoland sheep, EFM–LAC: East Friesian milk and Lacaune sheep and crosses of both breeds.

In all genotyped samples from 17 MV affected flocks, the putative protective *CCR5* promoter deletion was observed at a relatively low frequency of about 16%. Its frequency was even lower in the breed subsets EFM–LAC (10%) and TEX-x (11%), whereas it was higher in MLS-x (21%) and GBM (40%). No deviation from the Hardy–Weinberg equilibrium was found for *CCR5* genotype frequencies in all samples and in breed subsets.

In all sheep as well as in the breed subsets TEX-x and EFM–LAC, the putative protective allele (del), as well as the putative protective genotype (del/del) of the *CCR5* promoter occurred at higher frequencies in the MV negative compared to the MV positive sheep (Table 2). However, the opposite situation was observed in the breed subsets GBM and MLS-x, where this allele and genotype were observed more frequently in MV positive than MV negative sheep. The differences in allele frequencies between MV negative and positive samples were statistically significant in all samples, but not in any of the breed subsets. In all samples and in all breed subsets, no significant association with the serological MV status was found for the putative protective and risk genotypes of the *CCR5* promoter (Table 2).

**Table 2:** ***CCR5*** **promoter variant (wild type/deletion) allele and genotype frequencies in serologically MV positive and negative sheep**

Breed subset	MV status	*CCR5* allele frequency (n chromosomes)		*P* value	*CCR5* genotype frequency		*P* value
(n sheep)	(n sheep)				(n sheep)	(n sheep)	
		del	wt		del/del	del/wt, wt/wt	
All (521)	Negative (206)	0.19 (77)	0.81 (335)	0.021	0.04 (8)	0.96 (198)	0.519
	Positive (315)	0.13 (85)	0.87 (545)		0.03 (9)	0.97 (306)	
TEX-x (337)	Negative (96)	0.14 (26)	0.86 (166)	0.177	0.02 (2)	0.98 (94)	0.321
	Positive (241)	0.10 (49)	0.90 (433)		0.01 (2)	0.99 (239)	
GBM (39)	Negative (35)	0.37 (26)	0.63 (44)	0.253	0.11 (4)	0.89 (31)	0.321
	Positive (4)	0.63 (5)	0.37 (3)		0.25 (1)	0.75 (3)	
MLS-x (125)	Negative (62)	0.17 (21)	0.83 (103)	0.135	0.02 (1)	0.98 (61)	0.114
	Positive (63)	0.25 (31)	0.75 (95)		0.10 (6)	0.90 (57)	
EFM–LAC (20)	Negative (13)	0.15 (4)	0.85 (22)	0.278	0.08 (1)	0.92 (12)	1.000
	positive (7)	0.00 (0)	1.00 (14)		0.00 (0)	1.00 (7)	

del: deletion, wt: wild type, TEX-x: purebred and crossbred German Texel sheep, GBM: purebred German Blackheaded Mutton sheep, MLS-x: purebred and crossbred Merinoland sheep, EFM–LAC: East Friesian milk and Lacaune sheep and crosses of both breeds.

### Median MV ELISA S/P values of sheep with and without putative risk alleles of *TMEM154* and *CCR5*

**Figure 1:**
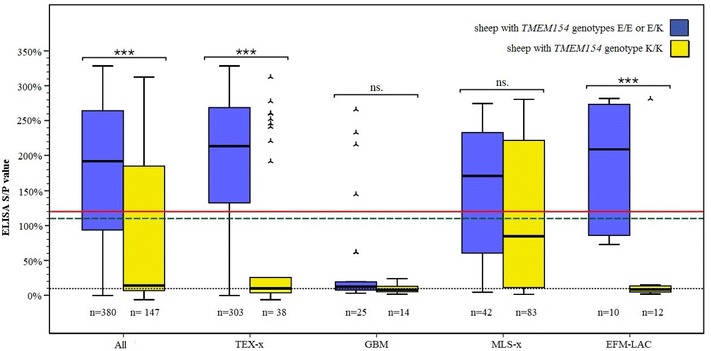
**Box plots depicting MV ELISA S/P values in sheep carrying genotypes with (blue) and without (yellow) the putative** ***TMEM154*** **risk allele (E), in all sheep and in breed subsets.** P values are resulting from nonparametric analyses comparing median MV ELISA S/P values of groups. ***P < 0.001, *ns* not significant (*P* > 0.05). The black dotted line indicates the median ELISA S/P value of sheep from six serologically MV negative flocks (about 9%). Sheep with ELISA S/P values below 110% (green dashed horizontal line) were considered serologically MV negative. Sheep with ELISA S/P values over 120% (red solid line) were considered serologically MV positive. TEX-x: purebred and crossbred German Texel sheep, GBM purebred German Blackheaded Mutton sheep, MLS-x: purebred and crossbred Merinoland sheep, EFM–LAC: East Friesian milk and Lacaune sheep and crosses of both breeds.

For *TMEM154*, in all sheep and in all breed subsets, except for GBM, the median ELISA S/P values of sheep with risk allele (*TMEM154* genotypes EE and EK) were higher than the cut-off value (Figure 1, solid red line) and varied around 190%. In contrast, in all sheep and in all breed subsets, the median ELISA S/P values of sheep without risk allele (carrying the genotype KK) were lower than the cut-off value (Figure 1). In all sheep and in subsets TEX-x and EFM–LAC, the median ELISA S/Ps of sheep with the protective genotype (KK) were lower compared to the putative risk genotypes (EE or EK). In detail, in all samples and in TEX-x and EFM–LAC breed subsets, the median of S/P values from sheep with the protective genotype (KK) were 13.76, 21.69 and 25.11 fold lower than those of sheep with risk genotypes (EK or EE). These differences were not significant for the breed subsets GBM and only approached statistical significance (*P* = 0.077) in MLS-x.

The median ELISA S/P value of sheep from the 6 MV negative flocks is indicated in Figure 1 (black dotted line). Interestingly, this median ELISA S/P value was not significantly different (pairwise comparisons revealed *P* > 0.05) from those of sheep from the subsets GBM, EFM–LAC and TEX-x, carrying no *TMEM154* risk allele. In contrast, a noticeably high median ELISA S/P value (84.67%) was observed in sheep from the subset MLX-x with this genotype (KK). It differed significantly (*P* values of pairwise comparisons ranged from 0.023 to < 0.001) from those of sheep with the same genotype from other subsets as well as from those of sheep from negative flocks.

Concerning sheep with and without *CCR5* risk allele (promoter deletion), the differences in median ELISA S/P values in all sheep and in breed subsets did not reach statistical significance in any group (Additional file 4).

### Relative risk to be serologically positive for sheep with and without putative risk alleles of *TMEM154* and *CCR5*

The relative risk to be serologically MV positive was calculated for sheep carrying one or two copies of the putative risk allele (*TMEM154*: E at position 35, *CCR5*: wild type promoter sequence), and compared to that of sheep carrying no risk allele. In the breed subgroups GBM and EFM–LAC, zero observations in serologically MV positive or negative groups produced large confidence intervals. Therefore, this analysis was only done in all sheep and for the two larger breed subsets, TEX-x and MLS-x (Table 3). In all samples and in the breed subgroup TEX-x, sheep carrying at least a single copy of the *TMEM154* risk allele had a significantly higher risk to be serologically MV positive than sheep with the KK genotype (relative risk of 2.26 and 3.30, respectively). Within the breed subset MLS-x, the relative risk to be serologically MV positive for sheep carrying one or two copies of the *TMEM154* risk allele was only 1.38. Moreover, the *P* value narrowly missed the significance threshold in this breed subset (Table 3).

**Table 3:** **Relative risk of infection in sheep with one or two copies of the** ***TMEM154*** **E allele**

Breed subset	Parameters	*TMEM154* genotypes
		EE, EK vs. KK
All	Relative risk	2.255
	95% CI	1.767–2.878
	*P* value	< 0.001
TEX-x	Relative risk	3.302
	95% CI	1.860–5.862
	*P* value	< 0.001
MLS-x	Relative risk	1.389
	95% CI	0.991–1.945
	*P* value	0.056

CI: confidence interval, TEX-x: purebred and crossbred German Texel sheep, MLS-x: purebred and crossbred Merinoland sheep.

A significant difference in the relative risk to be serologically MV positive for sheep with one or two copies of the *CCR5* promoter wild type allele, compared to sheep without this putative risk factor, was only observed for the breed subset MLS-x. However, in this subset, the risk for sheep to be serologically positive with one or two copies of the putative risk allele was about half (0.56-fold) of that of sheep without this allele (Additional file 5).

## Discussion

This study aimed at evaluating the association of sequence variants in two candidate genes for MV susceptibility (*TMEM154* and *CCR5*) with serological MV infection status (cut-off-based classification) as well as with ELISA values (S/P ratio) in German sheep flocks. For this purpose, samples were collected from sheep flocks already known, or suspected to be MV affected, without considering their breed background. It was remarkable but not unexpected that the great majority (15 out of 17) of the MV positive flocks contained purebred and/or crossbred sheep of the breed Texel (TEX-x), allowing clustering of these samples into the largest breed subset for following analyses. This high proportion of MV positive TEX-x flocks among the sampled flocks may be explained on the one hand by the fact that Texel is the dominating breed in commercial sheep flocks in Northern Germany, where most flocks for this study were sampled. On the other hand, it should also be taken into consideration that the Texel breed is known for high MV susceptibility, based on both seroconversion rates and number of sheep showing clinical signs in an infected flock [13]. In the past it was suspected that this cosmopolitan breed might be responsible for the introduction of MV into some previously MV-free countries, e.g. Great Britain [11, 13]. One of the other breed subsets of this study included sheep from two milk sheep breeds, East Friesian milk and Lacaune, and their crosses (EFM–LAC). In Germany, the breeds Texel and East Friesian milk are targeted in regional or federal MV monitoring and eradication programs (e.g., via serological testing and culling). Also in other countries, East Friesian milk sheep were observed to be higher susceptible to MV than other breeds [12]. In a German study on MV seropositive sheep of the breeds Texel, Finnsheep, Ile the France and Merinoland, sheep of all breeds except for Merinoland developed clinical signs of disease [13].

In all samples from 17 MV affected German sheep flocks, both crosstab and non-parametric analyses showed a significant association between the amino acid substitution at position 35 of *TMEM154* and both the serological MV status (cut-off-based classification) and the median ELISA S/P value. More precisely, the *TMEM154* genotype KK was associated with a lower number of serologically MV positive sheep and with a lower median ELISA S/P value, whereas for sheep with one or two copies of the E allele, the situation was reversed. The same was observed for the breed subsets, partly with an even more explicit effect of the genotype KK. However, this was not statistically significant in the breed subsets GBM and MLS-x. For the GBM subset, the missing significant association can be explained by the very low number of serologically positive sheep (four out of 39 sheep). It is of interest that these purebred GBM sheep were kept together with purebred TEX sheep (Additional file 2, flocks 16b and 16a, respectively). Among the TEX sheep of this flock, a noticeably higher proportion (65%) was serologically MV positive. As sheep of similar ages were compared and kept in the same, relatively small flock, different environmental factors can be neglected. Hence, the different genetics of the two breeds, e.g., different frequencies of protective or risk alleles, is the most likely reason for the observed difference in the proportion of serologically MV positive sheep. In fact, the frequency of sheep carrying the *TMEM154* genotype KK was 40% among the GBM and 0% among the TEX sheep of this flock.

In the breed subset MLS-x, the association of *TMEM154* E/K with serological MV status and median ELISA S/P value was not significant either but approached the significance threshold. A remarkably high frequency of MV seropositive sheep with the genotype KK was observed in this breed subset (47%), compared to 24% in TEX-x, 8% in EFM–LAC and 0% in GBM. This was also illustrated by a notably higher median ELISA S/P value in sheep with the KK genotype of this subset compared with the other breed subsets as well as with the median ELISA value of MV negative flocks (Figure 1). It has been shown in other studies that the KK genotype is not fully protective. In a previous study [19], about 26% of sheep carrying two K alleles were seropositive. Several factors may influence the proportion of seropositive sheep with this genotype in a MV positive flock, such as viral dose, route of infection, additional host risk factors (e.g., animal crowding, other breed-specific genetic factors), presence of a distinct and/or more than one SRLV strain(s) or subtype(s), and coinfection with another disease at the same time [19, 20, 34]. Sider et al. [34] reported different odds of infection for sheep carrying the same *TMEM154* E35K genotypes, but infected with different SRLV genotypes, postulating that not only the host but also the SRLV genotype affects the relative risk of infection in sheep. Hence, an (additional) infection with an SRLV strain or subtype that is different from the virus strain(s) or subtype(s) circulating in the other analyzed flocks could be the reason for the higher percentage of seropositive sheep with the KK genotype in the MLS-x flock compared to the other subsets. Until now, no information has been available on virus subtypes or strains circulating in the German sheep population. In a follow-up study, the samples collected in this study, together with additional samples from other German regions, should be used for genotyping and characterization of virus strains. On the one hand, this would make it possible to test for a virus subtype-dependent association of host gene variants with susceptibility or resistance to infection. On the other hand, this is a prerequisite to establish more precise tools for phenotyping the MV infection status of the German sheep population.

In all samples, sheep with one or two copies of the E allele of *TMEM154* had a relative risk of 2.26 to be serologically MV positive. This is comparable to another study [19] in which the relative risk of infection for sheep carrying one or two copies of the *TMEM154* risk haplotypes (haplotypes including E at position 35) varied from 1.27 to 5.30 between cohorts (populations with different breed background, age at sampling and seroprevalence) with an overall relative risk of 2.85.

In this study, the deletion in the *CCR5* promotor did not show a consistent association with serological MV status or median ELISA S/P values. The main reason might be the low number of sheep carrying two copies of the *CCR5* promoter deletion (only 17 out of 521 genotyped sheep). For sheep carrying one or two copies or the *CCR5* promoter wild type, a significant difference in the relative risk to be serologically MV positive compared to sheep with the promoter deletion was only found in the breed subgroup MLS-x. Unexpectedly, in this subset, the relative risk was higher for sheep carrying two copies of the putative protective allele (deletion) than for sheep carrying one or two copies of the putative risk allele (wild type). Hence, the direction of the association was opposed to that observed in a US sheep flock originating from Idaho [18], but consistent with results in a US sheep flock from Iowa [20]. It should be reconsidered that in the study of White et al. [18], a different phenotype was used for association analysis with *CCR5* (proviral load). It is possible that different susceptibility phenotypes (here antibody response and provirus load) are controlled in part or completely by different host genes. At this point it should be mentioned that, up to our knowledge, the amino acid mutation at position 35 of *TMEM154* is the only genetic marker displaying association with both the serological status as well as the proviral level regarding MV infection in sheep [19, 20].

It is also possible that the deletion in the *CCR5* promoter is not directly influencing the analyzed phenotype(s), but is linked with an unknown causal genetic variant. In such a case, the allele which is linked with the causal protective or risk allele can vary between breeds and populations.

The present study was based on the collection of samples in commercial German sheep flocks which were tested for MV infection status by a common and approved serological method. The median ELISA S/P value of samples from 6 MV negative flocks was 9.69% with a non-conservative approximate 95% confidence interval (95% CI) of 1.78–52.30%. Hence, the recommended cut-off value for the used ELISA (≤ 110 negative, ≥ 120 positive) is approximately twofold the upper limit of the confidence interval of the negative flocks. Therefore, it is possible that some sheep which were classified as serologically MV negative or doubtful in fact were infected.

A further limitation of this study may be the well-known high genetic variability of the small ruminant lentivirus, which is promoted by high mutation and recombination rates during viral replication [35]. Therefore, the diagnostic power of a MV ELISA can be limited by the virus subtype(s) or strain(s) present in a flock. The MV ELISA used in the present study is one of three ELISAs which are approved for determination of SRLV infection in sheep and goats by the German licensing authority. In a comparison test including these three and also other ELISAs used in other countries, the proportion of samples from sheep with clinical MV symptoms detected as serologically positive varied between the different ELISAs (Günter Kotterba, German Friedrich-Loeffler-Institut (FLI), Federal Research Institute for Animal Health, Island of Riems, Germany, personal communication). This may be due to differences in the sensitivity of ELISAs for certain virus strains or genotypes. For instance, correct identification of SRLV strain E, which is present in Italy and highly divergent from other SRLV strains, strain-specific antigens had to be developed and tested [36]. In a study on SRLVs in Jordan, where no information on present SRLV genotypes was available, the maximum of positive samples was found using three different ELISA kits [37].

It can be concluded that the amino acid substitution at position 35 of *TMEM154* is a promising marker for breeding towards a lower MV susceptibility (in terms of a lower number of serologically positive sheep) in Germany, at least in flocks based on the Texel breed, while this remains questionable for the deletion in the *CCR5* promoter. The presence of the protective *TMEM154* variant in diverse sheep breeds all over the world indicates that it is an old mutation and carried in a most likely quite short haplotype. Therefore, the risk of associated negative effects on other selection traits will be low. However, this has to be monitored carefully during selection.

In consecutive analyses, the findings of this study should be verified and possibly enlarged by including a higher number of MV affected flocks of other breeds and employing additional phenotyping tools, e.g., other MV ELISA kits and methods for the measurement of provirus load. In particular, a follow-up study employing genome-wide association analysis in a higher number of samples from MV affected Merinoland sheep might be able to identify additional genetic variants with a breed-specific impact on MV susceptibility.

## Additional files

**Additional file 1.** **Primers used for genotyping and sequencing.**

**Additional file 2.** **Origin (German state), breed composition, percentage of serologically MV-positive samples and classification into breed subset for each sampled sheep flock.**

**Additional file 3.** **Numbers of serologically MV negative and positive sheep and description of MV ELISA S/P values (%) in all 17 MV affected flocks and within breed subsets.**

**Additional file 4.** **Box plots depicting MV ELISA S/P values in sheep carrying genotypes with (blue) and without (yellow) the putative** ***CCR5*** **promoter risk allele (wt), in all sheep and in breed subsets.** *P* values are resulting from nonparametric analyses comparing median MV ELISA S/P values of groups. ns: not significant (*P* > 0.05). The black dotted line indicates the median ELISA S/P value of sheep from six serologically MV negative flocks (about 9%). Sheep with ELISA S/P values below 110% (green dashed horizontal line) were considered serologically MV negative. Sheep with ELISA S/P values over 120% (red solid line) were considered serologically MV positive. wt: wild type; del: deletion; TEX-x: purebred and crossbred German Texel sheep; GBM: purebred German Blackheaded Mutton sheep; MLS-x: purebred and crossbred Merinoland sheep; EFM-LAC: East Friesian Milk and Lacaune sheep and crosses of both breeds.

**Additional file 5.** **Relative risk of infection in sheep with one or two copies of the** ***CCR5*** **promoter deletion.**

## Abbreviations

*CCR5*: chemokine (C-C motif) receptor type 5
EFM: East Friesian milk
ELISA: enzyme-linked immunosorbent assay
GBM: German Blackheaded Mutton sheep
LAC: Lacaune sheep
MLS: Merinoland sheep
MV: maedi-visna
OD: optical density
PCR: polymerase chain reaction
S/P: ratio between mean OD of sample and mean OD of positive control
SRLV: small ruminant lentiviruses
TEX: German Texel sheep
*TMEM154*: transmembrane protein 154
