# Supplementary material for: First survey on association of TMEM154 and CCR5 variants with serological maedi-visna status of sheep in German flocks
Source: Vet Res. 2018 Apr 19;49:36. doi: 10.1186/s13567-018-0533-y (PMC5909245; doi:10.1186/s13567-018-0533-y)
Supplement: Supplementary file 1 — Additional file 1. Primers used for genotyping and sequencing. [file 13567_2018_533_MOESM1_ESM.docx]

| Gene | primer sequences | product size | purpose |
| --- | --- | --- | --- |
| *TMEM154* | 5'- CCACAGGAGAGGAGRACACA-3' (forward) | 40/41 bp | determination of nucleotide substitution NC_019474.2:g.4860407G>A, resulting in amino acid substitution E35K, using KASP technology (LGC, Hoddesdon, UK) |
|  | 5'-GGGCACGTCTCCTGACAGTT**T**-3' (reverse, FAM-labeled, K allele) |  |  |
|  | 5'-GGCACGTCTCCTGACAGTT**C**-3' (reverse, HEX-labeled, E allele) |  |  |
| *TMEM154* | 5'-GCTAGACACTGCCAAGCTTC-3' (forward) | 788 bp | amplification and sequencing for verification of genotyping results |
|  | 5'-TGTCACTGAAACAAGTCATCACT-3' (reverse) |  |  |
| *CCR5* | 5'-CCCCATTGATAAGCCCTACA-3' (forward) | 160/156 bp | determination of promotor region deletion NC_019476.2:g.52961717_52961714delAATG (minus strand); amplification and sequencing for verification of genotyping results |
|  | 5'-CACCCAACTACCCAAATGGT-3'  (reverse, FAM-labeled or unlabeled) |  |  |

Bold letters: nucleotide positions leading to allele specific binding of primers.
