# Supplementary material for: First survey on association of TMEM154 and CCR5 variants with serological maedi-visna status of sheep in German flocks
Source: Vet Res. 2018 Apr 19;49:36. doi: 10.1186/s13567-018-0533-y (PMC5909245; doi:10.1186/s13567-018-0533-y)
Supplement: Supplementary file 2 — Additional file 2. Origin (German state), breed composition, percentage of serologically MV-positive samples and classification into breed subset for each sampled sheep flock. [file 13567_2018_533_MOESM2_ESM.docx]

| Flock no. | state | Breed background of flocks | sampled sheep (n)^1^ | serologically MV positive (%) | breed subset |
| --- | --- | --- | --- | --- | --- |
| 1 | SH | TEX-x | 33 | 60.60 | 1 |
| 2 | SH | TEX-x | 16 | 68.75 | 1 |
| 3 | SH | TEX-x | 26 | 57.69 | 1 |
| 4 | SH | TEX-x | 41 | 73.17 | 1 |
| 5 | SH | TEX-x | 11 | 100.00 | 1 |
| 6 | SH | TEX-x | 30 | 76.67 | 1 |
| 7 | SH | TEX-x | 21 | 66.66 | 1 |
| 8 | SH | TEX-x | 22 | 77.27 | 1 |
| 9 | SH | TEX-x | 30 | 83.33 | 1 |
| 10 | SH | TEX-x | 11 | 63.64 | 1 |
| 11 | SH | TEX | 19 | 78.95 | 1 |
| 12 | SH | TEX | 5 | 80.00 | 1 |
| 13 | SH | EFM | 6 | 0.00 | - |
| 14 | NRW | TEX | 21 | 80.95 | 1 |
| 15 | NRW | TEX-x | 15 | 73.33 | 1 |
| 16.a | NRW | TEX | 43 | 65.11 | 1 |
| 16.b | NRW | GBM | 39 | 10.26 | 2 |
| 17 | NRW | TEX, EFM | 17 | 0.00 | - |
| 18 | NRW | RHO | 25 | 0.00 | - |
| 19 | NRW | Mix of several breeds | 17 | 0.00 | - |
| 20 | NRW | SUF | 32 | 0.00 | - |
| 21 | MVP | RPL | 18 | 0.00 | - |
| 22 | HE | MLS-x | 125 | 50.40 | 3 |
| 23 | BW | EFM, LAC, EFM-LAC | 22 | 36.36 | 4 |

^1^ without sheep with suspicious ELISA S/P results (*n*= 11).

Abbreviation of German states: SH (Schleswig-Holstein), NRW (Nordrhein-Westfalen), MVP (Mecklenburg-Vorpommern), HE (Hessen), BW (Baden-Württemberg).

Abbreviation of breeds: -x (not all sheep were purebred), TEX (German Texel), EFM (East Friesian Milk sheep), GMB (German Blackheaded Mutton), RHO (Rhoen sheep), SUF (Suffolk), RPL (Rough-coated Pomeranian Landrace), MLS-x (Merinoland sheep), LAC (Lacaune).

Breed subsets: 1 = purebred and crossbred German Texel (TEX-x), 2 = purebred German Blackheaded Mutton (GBM), 3 = purebred and crossbred Merinoland sheep (MLS-x), 4 = East Friesian Milk and Lacaune sheep and crosses of both breeds (EFM-LAC).
