# Supplementary material for: First survey on association of TMEM154 and CCR5 variants with serological maedi-visna status of sheep in German flocks
Source: Vet Res. 2018 Apr 19;49:36. doi: 10.1186/s13567-018-0533-y (PMC5909245; doi:10.1186/s13567-018-0533-y)
Supplement: Supplementary file 3 — Additional file 3. Numbers of serologically MV negative and positive sheep and description of MV ELISA S/P values (%) in all 17 MV affected flocks and within breed subsets. [file 13567_2018_533_MOESM3_ESM.docx]

| Breed subset | MV status (n) | MV ELISA S/P value range | median MV ELISA S/P values | quartile of MV ELISA S/P values  Q1, Q3 | *p* value  (test of normal distribution) |
| --- | --- | --- | --- | --- | --- |
|  |  | min, max |  |  |  |
| All | negative (207) | -6.34, 109.65 | 12.95 | 5.56, 37.37 | <0.001 |
|  | positive (323) | 121.89, 328.63 | 245.89 | 182.27, 270.72 | < 0.001 |
| TEX-x | negative (96) | -6.34, 109.65 | 13.67 | 5.44, 42.07 | < 0.001 |
|  | positive (248) | 123.11, 328.63 | 246.70 | 183.07, 273.29 | < 0.001 |
| GMB | negative (35) | 1.61, 61.92 | 9.14 | 5.19, 13.60 | < 0.001 |
|  | positive (4) | 144.65, 266.04 | 224.51 | 162.48, 257.80 | 0.681 |
| MLS-x | negative (62) | 1.45, 104.05 | 14.14 | 6.47, 59.03 | < 0.001 |
|  | positive (63) | 122.10, 280.70 | 233.17 | 176.20, 263.39 | < 0.001 |
| EFM-LAC | negative (14) | 1.80, 85.84 | 10.25 | 4.98, 29.42 | < 0.001 |
|  | positive (8) | 121.89, 282.01 | 269.10 | 183.38, 280.92 | 0.002 |

TEX-x: purebred and crossbred German Texel sheep; GBM: purebred German Blackheaded Mutton sheep; MLS-x: purebred and crossbred Merinoland sheep; EFM-LAC: East Friesian Milk and Lacaune sheep and crosses of both breeds.
