# Supplementary figures and images for: First survey on association of TMEM154 and CCR5 variants with serological maedi-visna status of sheep in German flocks
Source: Vet Res. 2018 Apr 19;49:36. doi: 10.1186/s13567-018-0533-y (PMC5909245; doi:10.1186/s13567-018-0533-y)

## Slide 1
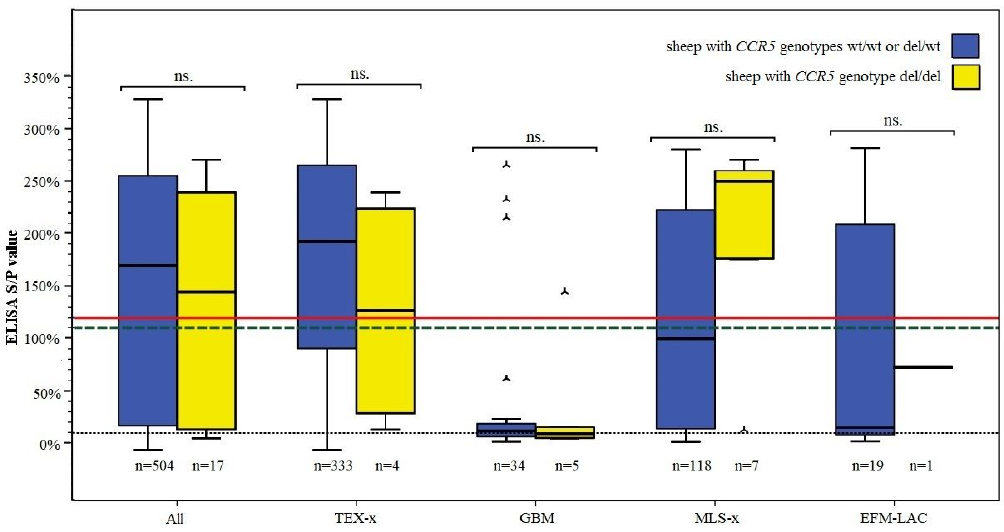

Supplement: Supplementary file 4 — Additional file 4. Box plots depicting MV ELISA S/P values in sheep carrying genotypes with (blue) and without (yellow) the putative CCR5 promoter risk allele (wt), in all sheep and in breed subsets. P values are resulting from nonparametric analyses comparing median MV ELISA S/P values of groups. ns: not significant (P > 0.05). The black dotted line indicates the median ELISA S/P value of sheep from six serologically MV negative flocks (about 9%). Sheep with ELISA S/P values below 110% (green dashed horizontal line) were considered serologically MV negative. Sheep with ELISA S/P values over 120% (red solid line) were considered serologically MV positive. wt: wild type; del: deletion; TEX-x: purebred and crossbred German Texel sheep; GBM: purebred German Blackheaded Mutton sheep; MLS-x: purebred and crossbred Merinoland sheep; EFM-LAC: East Friesian Milk and Lacaune sheep and crosses of both breeds. [file 13567_2018_533_MOESM4_ESM.pptx]
