# Supplementary material for: First survey on association of TMEM154 and CCR5 variants with serological maedi-visna status of sheep in German flocks
Source: Vet Res. 2018 Apr 19;49:36. doi: 10.1186/s13567-018-0533-y (PMC5909245; doi:10.1186/s13567-018-0533-y)
Supplement: Supplementary file 5 — Additional file 5. Relative risk of infection in sheep with one or two copies of the CCR5 promoter deletion. [file 13567_2018_533_MOESM5_ESM.docx]

| Breed subset | parameters | *CCR5* genotypes |
| --- | --- | --- |
|  |  | wt/ wt, del/wt vs. del/del |
| All | relative risk | 1.146 |
|  | 95% CI | 0.728 - 1.805 |
|  | *p* value | 0.554 |
| TEX-x | relative risk | 1.435 |
|  | 95% CI | 0.537 - 3.833 |
|  | *p* value | 0.471 |
| MLS-x | relative risk | 0.563 |
|  | 95% CI | 0.395 - 0.804 |
|  | *p* value | 0.001 |

del: deletion; wt: wild type; CI: confidence interval; TEX-x: purebred and crossbred German Texel sheep; MLS-x: purebred and crossbred Merinoland sheep.
